# Fatigued with Friedewald: why isn't everyone onboard yet with the new LDL-C equations?

**DOI:** 10.3389/fcvm.2025.1534460

**Published:** 2025-02-27

**Authors:** Madhusudhanan Narasimhan, Jing Cao, Jeffrey W. Meeusen, Alan T. Remaley, Seth S. Martin, Alagarraju Muthukumar

**Affiliations:** ^1^ Department of Pathology, University of Texas Southwestern Medical Center, Dallas, TX, United States; ^2^ Chemistry and Metabolic Disease Laboratory, Children’s Medical Center, Dallas, TX, United States; ^3^ Department of Laboratory Medicine and Pathology, Mayo Clinic, Rochester, MN, United States; ^4^ Lipoprotein Metabolism Laboratory, Translational Vascular Medicine Branch, National Heart, Lung, and Blood Institute, National Institutes of Health, Bethesda, MD, United States; ^5^ Ciccarone Center for the Prevention of Cardiovascular Disease, Johns Hopkins School of Medicine, Baltimore, MD, United States

**Keywords:** Friedewald equation, Martin/Hopkins equation, NIH-Sampson LDL-C equation, direct LDL-C measurement, LDL-C equations

Atherosclerotic cardiovascular disease (ASCVD) continues to be the leading cause of death among chronic diseases, with its prevalence rapidly rising in many countries. Among several factors, hypertension and dyslipidemia are well-established risk factors for ASCVD. Numerous epidemiologic, experimental, genetic, and randomized placebo-controlled clinical trials have firmly established a causal relationship between low-density lipoprotein cholesterol (LDL-C) and ASCVD (1). An accurate assessment and reporting of LDL-C is, therefore, pivotal for dyslipidemia diagnosis and management, especially for the LDL-C goal-based ASCVD primary and secondary prevention treatment decisions endorsed by various national and international organizations (2, 3). An LDL-C level ranging from 70 to 99 mg/dl is considered a reasonable target for primary prevention, leading to the use of moderate-intensity statin therapy (4). Recently, in very high-risk categories of ASCVD, the European Society of Cardiology (ESC) has recommended a stringently low target level of <55 mg/dl for both primary and secondary prevention and even <40 mg/dl in selected patients with recurrent ASCVD (5). The Friedewald (FW) equation, used worldwide since 1972 for LDL-C calculation due to its simplicity and ease of implementation, is well known to have accuracy issues, particularly, at TG levels ≥150 mg/dl and when combined with non-HDL-C levels <100 mg/dl (6). With more than 200 million patients on lipid-lowering drugs like statins globally, and the introduction of new drug classes like PSCK9 inhibitors that can drastically lower LDL-C levels below 50 mg/dl, the limitations of FW LDL-C are increasingly becoming apparent. Recent guideline changes from fasting to non-fasting states for initial dyslipidemia screening and increased reliance on reflexive direct LDL-C testing, further challenges the clinical utility of the FW equation.

Many laboratories utilize direct LDL-C assays as part of a reflex protocol at TG ≥400 mg/dl. Despite the cost associated with the direct LDL-C testing in place of freely available calculations, some laboratories still prefer direct LDL-C assay as part of lipid panel. This approach is followed regardless of the patient's LDL-C or TG levels. However, Miller et al. have shown biases with direct LDL-C assays, often leading to the overestimating of LDL-C in dyslipidemia patients (7). Importantly, their analytical performance in the current context of potent lipid-lowering therapies and stringent LDL-C target levels have remained insufficiently evaluated. It is notable that direct LDL-C assays are also susceptible to discrepancies due to different methodologies and inherent variations in composition of low-density lipoproteins in different diseases. There remains a challenge for the Lipid Standardization program to standardize LDL-C assays.

Many alternative LDL-C formulae to the FW equation have been developed over the years. The first alternative equation that clearly showed an advantage over FW was developed by Martin et al. in 2013 [Martin-Hopkins (MH) equation], which replaced the fixed TG denominator of 5 in FW with an empirically determined VLDL-C: TG ratio from each individual's lipid profile based on a table of TGs and non-high-density lipoprotein cholesterol (non-HDL-C) concentrations by analyzing 1,350,908 patients based on the vertical auto profile (VAP) method (8). Importantly, the findings of Martin et al. demonstrated that the FW equation not only underperforms at TG ≥400 mg/dl but also at TG levels as low as 150 mg/dl. This equation demonstrated a strong correlation with reference method and outperformed the FW equation in accuracy, which was independently confirmed by many external studies, including multiple studies measuring LDL-C by the beta-quantification reference method (9). This has led to the invocation of Class IIa recommendation of MH's equation for the use of a “modified estimation” of LDL-C in patients with LDL-C <70 mg/dl and TG levels ≥150 mg/dl by the 2018 AHA/ACC/multi-society cholesterol guideline. A modified version of the equation called the extended MH equation has recently demonstrated improved LDL-C accuracy in patients with TG levels beyond 400 mg/dl and up to 800 mg/dl. Its strength lies in its inclusion of large validation and derivation data sets with individuals who are representative of the US population (10).

Meanwhile, Sampson et al. derived a novel LDL-C equation to improve the accuracy of LDL-C estimation using 18,715 samples from 8,656 patients (the NIH database) with TG values up to 800 mg/dl. This employed a non-linear least square regression analysis to estimate VLDL-C using TG and non-HDL-C as in the MH equation (11). The new NIH equation was verified with over 250,000 patient samples from LabCorp and other clinical laboratories and demonstrated to be more accurate in the calculation of LDL-C compared to the FW equation. Like the FW equation, this new equation is based on the beta-quantification reference method for LDL-C, which is used by the CDC to standardize all LDL-C methods, including the direct assays. In addition, a more recent and improvised version of original Sampson equation called as the “enhanced Sampson-NIH equation” is also available by incorporating apolipoprotein B as one of the independent variables (12).

Although the newer LDL-C equations have been independently verified to calculate LDL-C up to TG 800 mg/dl with reasonable accuracy compared with direct LDL-C measurements in different populations (13–15), at TG levels ≥500 mg/dl, the clinical priority, in these cases, should be to first reduce the elevated TG level to prevent acute pancreatitis. Given this clinical need and the relatively better performance of the new calculations-based LDL-C estimations at even higher TG levels (Sampson-NIH, up to 800 mg/dl) and lower LDL-C levels (Martin/Hopkins equation, LDL-C <70 mg/dl), it is apparent that fewer patients will only require reflexive direct LDL-C measurements. However, it must be noted that no calculation method is perfect, and direct measurement may still be necessary in some cases, particularly when precise quantification of very low LDL-C levels is critical for clinical decision-making. Notably, in case of rare lipid disorders as in type III hyperlipidemia, the FW equation performs poorly due to inaccurate VLDL-C estimation. However, the Martin/Hopkins equation uses an adjustable factor for the TG: VLDL-C ratio, which may provide more accurate estimates in atypical lipid profiles. Nevertheless, they may still have limitations in accurately estimating LDL-C in type III hyperlipidemia due to the condition's unique lipid profile and an accumulation of remnant lipoproteins. Also, in case of post-prandial conditions, unlike the FW equation, which is limited to fasting samples, the newer equations perform equally well in both fasting and non-fasting samples. Thus, while these newer equations offer improvements over the FW equation and can largely curtail the need for reflexive direct LDL-C measurements, they may not eliminate it entirely, especially for very high TG levels (>800 mg/dl), extremely low levels of LDL-C (below 40 mg/dl), or rare lipid disorders. However, it is important to know that direct LDL-C measurements can also be discordant, particularly in patients with high cardiovascular risk and/or dyslipidemia (7). Table 1 summarizes different LDL-C estimation methods comparison.

**Table 1:** From direct to derived: comparison of LDL-C estimation strategies.

Method	Methodological approach	Pros	Cons
Direct measurement	• Selectively precipitates non-LDL particles (VLDL-C and chylomicrons) to expose and measure LDL-C	• Assays are automated; Enables high throughput	• Adds to cost and time
		• Performs well in fasting and non-fasting samples	• Hemolysis, sample mishandling, and remnant lipoproteins impact result accuracy
	• Uses homogeneous enzymatic colorimetric procedure	• Reliable at TGs above 400 mg/dl	• Lacks inter-laboratory standardization
		• Outperforms FW and allows better monitoring of CV disease risk	• Bias at either extremes of TG levels
Friedewald	• $\begin{array}{r} \mathrm{LDL}\text{-}C=\mathrm{TC}-\mathrm{HDL}\text{-}C-\left(\frac{\mathrm{TG}}{5}\right) \end{array}$	• Widely used and accepted	• Uses fixed TG:VLDL of 5:1
		• Simple to calculate and thus, cost effective	• Less reliable when TG >150 mg/dl and in patients with type III or type I hyperlipoproteinemia
		• Reasonably accurate for normal lipid profiles	• Inaccurate at low LDL-C levels (<100 mg/dl)
		• Allows comparison with older research and incorporated into many clinical guidelines	• Requires fasting sample for accurate result
Martin/Hopkins	$\begin{array}{r} \mathrm{LDL}\text{-}C\;=\;\mathrm{TC}-\mathrm{HDL}\text{-}C\text{–}\left(\frac{\mathrm{TG}}{\mathrm{adjustable}\;\mathrm{factor}}\right) \end{array}$	• Uses adjustable TG: VLDL-C ratio based on individual TG and non-HDL-C levels	• More complex calculation
		• Accurate in fasting and non-fasting samples	• Requires adjustable factor
		• AHA/ACC recommends for LDL-C with <70 mg/dl and TG up to 400 mg/dl	• Not suitable for very high TGs (>400 mg/dl)
			• Can be less accurate in familial hypertriglyceridemia
NIH-Sampson	• $\begin{array}{r} \mathrm{LDL}\text{-}C\;=\;\left(\frac{\mathrm{TC}}{0.948}\right)-\left(\frac{\mathrm{HDL}\text{-}C}{0.971}\right)-\left[\begin{array}{c} \left(\frac{\mathrm{TG}}{8.56}\right)+\mathrm{TG}\;\times \;\\ \frac{\mathrm{non}\text{-}\mathrm{HDL}\text{-}C}{2,140}-\\ \left(\frac{{\mathrm{TG}}^{2}}{16,100}\right) \end{array}\right]-9.44 \end{array}$	• Valid across TG levels up to 800 mg/dl	• Relatively complex formula than FW equation
		• Derived from β-quantification gold standard, enhancing reliability	• Need more validation at low LDL-C values
		• Multi-society guidelines endorse NIH-Sampson for non-fasting lipid screening	• Does not account for genetic variations affecting lipid metabolism

ACC, American College of Cardiology; AHA, American Heart Association; apoA-I, apolipoproteinA-I; apoB, apolipoprotein B; CV, cardiovascular; FW, Friedewald; HDL-C, high density lipoprotein cholesterol; LDL-C, low density lipoprotein-C; non-HDL-C, non-high density lipoprotein cholesterol; TC, total cholesterol; TG, triglycerides.

Given that ADLM and other associations have also acknowledged the strength of newer equations, it is important to address why are we still stuck in the old era and still using the FW equation? Recent informal surveys while indicating a slight increase in adoption of the newer equations, the uptake remains still lukewarm. This raises critical questions: What are the barriers to implementing the new LDL-C equations? Why does the reluctance to move on from the FW equation persist? Is it driven by convenience or perceived complications?

We believe the primary reason lies in the convenience of sticking to routine practices and a strong reluctance to change, as the FW equation has been entrenched in clinical use for over 5 decades now. This indeed is also largely fueled by the absence of universal guidelines. Without a clear consensus about how new equations may benefit patients, practitioners and facilities may adopt “*if it's not broken, don't fix it*” approach and continue with established practices.

One of the factors hindering the implementation of new equations, particularly, the MH equation is the prevalent misconception that it is copyrighted and would require a licensing fee for its commercial use. However, it has been recently clarified that the MH equation is royalty-free, addressing this concern. Additionally, some confusion may linger about the term “free” in “royalty-free”, as it could be interpreted that it is free only from ongoing royalties but still require a significant one-time licensing fee or other specific licensing terms. Recently, Johns Hopkins University has abandoned the patent application to facilitate the use of their equation without intellectual property restrictions (16). Notably, this decision means that this formula is allowed for broader access and application and can be used freely or even modified without any licensing barriers. Thus, the previously misconstrued concern regarding the “no royalty-free” notion is no longer an issue for the transition to using MH equation. Furthermore, despite some concern about the complexity of implementing the MH equation, Johns Hopkins has made this equation more readily accessible in formats that can be seamlessly integrated into LIS and middleware systems like Data Innovations. Laboratories are encouraged to contact JHTT-Communications@jh.edu and smart100@jhmi.edu for assistance with this process (16). Indeed, they have gone a step further and willing to share line-by-line sample code with labs/health systems that are intending to implement their equation. This approach allows faster, more convenient implementation, with each step in the code visible and modifiable for adaptability across LIS platforms and lab protocols. Besides, it can foster collaboration and troubleshooting through peer-reviewed code snippets. Indeed, the MH equation's ease of implementation is exemplified by its increasing adoption in laboratories around the world. Large commercial laboratories have successfully implemented the MH equation and currently uses as part of their lipid panels: Quest Diagnostics (test codes: 7600, 91716, 92052, 92053, 14852, 92061, 19543) and Clinical Pathology Laboratories (test code: 4228). Health systems such as Northwestern and the entire Brazil has also implemented MH equation and currently, UTSW is in the process of implementing the MH equation as well (9, 17). Efforts are also underway to build MH equation into the EPIC system directly. Encouragingly, adoption of MH has been further supported by the European Atherosclerosis Society and European Federation of Clinical Chemistry, National Lipid Association, World Heart Federation, Polish Lipid Association, and Multi-Society Recommendations of Brazil.

Likewise, the NIH-Sampson equation is free from any licensing restrictions, removing legal or financial barriers to adoption. It is also easier to implement in laboratory systems because its formula does not depend on a look-up table and thus it can be directly implemented into all LIS systems by end users. Notably, LabCorp has integrated the LDL-C (Sampson's NIH) equation and now offers the LDL-C estimation with this calculation (result code, 012059) as part of their lipid panel with order code, 303756 (11). The Seattle Children's Hospital (Test code: LAB18), Mayo Medical reference laboratory (Test ID: CLDL1), and Standford Medicine (Test code: LCDPC, LPDC) also uses the Sampson-NIH equations. After five decades of reliance on the FW equation, the Canadian Society of Clinical Chemists officially recommended the nationwide adoption of the NIH-Sampson equation and since July 2022, laboratories across Canada have been transitioning from the FW method to the NIH-Sampson (18). Recent guidelines from Mexico (19), Poland (20), and the United Kingdom (21) have also recommended transiting from FW to the Sampson-NIH equation.

While these recent changes reflect the growing recognition of the value of the new LDL-C equations and there is now momentum to move towards these next-gen equations, the transition is still far from complete. Although it must be acknowledged that FW equation have served well for decades, its well-known negative bias contributes to the undertreatment of patients, which is a major issue with lipid-lowering therapy (22). Whatever the reason for its ongoing use, be it convenience, familiarity, and or the perceived complexity of change, it is becoming increasingly untenable to persist with the FW equation given the crucial role that LDL-C plays in the management of cardiovascular disease, a leading worldwide cause of death and morbidity. Indeed, in our view it is long past due to break “free” from the conservative mindset and adopt one of the newer “free” LDL-C equations, specifically the Martin/Hopkins or Sampson-NIH.
